# Supplementary material for: ComK2 represses competence development for natural transformation in Staphylococcus aureus grown under strong oxygen limitation
Source: Commun Biol. 2025 Oct 2;8:1416. doi: 10.1038/s42003-025-08816-z (PMC12491500; doi:10.1038/s42003-025-08816-z)
Supplement: Supplementary file 1 — Supplementary Information [file 42003_2025_8816_MOESM1_ESM.pdf]

Supplementary Information

ComK2 represses competence development for natural transformation in *Staphylococcus aureus* grown under strong oxygen limitation

Shi Yuan Feng [1,†], Yannis Arab [1,2,†], Yolande Hauck [1], Pierre Poirette [1,2], Magali Noiray [1], Sophie Quevillon-Cheruel [1], Stéphanie Marsin [1], Jessica Andreani [1] and Nicolas Mirouze [1,2,*]

[†] These authors contributed equally
[*] Corresponding author,
Email: nicolas.mirouze@inserm.fr

[1] Université Paris-Saclay, CEA, CNRS, Institute for Integrative Biology of the Cell (I2BC), 9198, Gif-sur-Yvette, France
[2] Université Paris-Saclay, UVSQ, Inserm, Infection et inflammation, Montigny-Le-Bretonneux, France.

Supplementary Figures

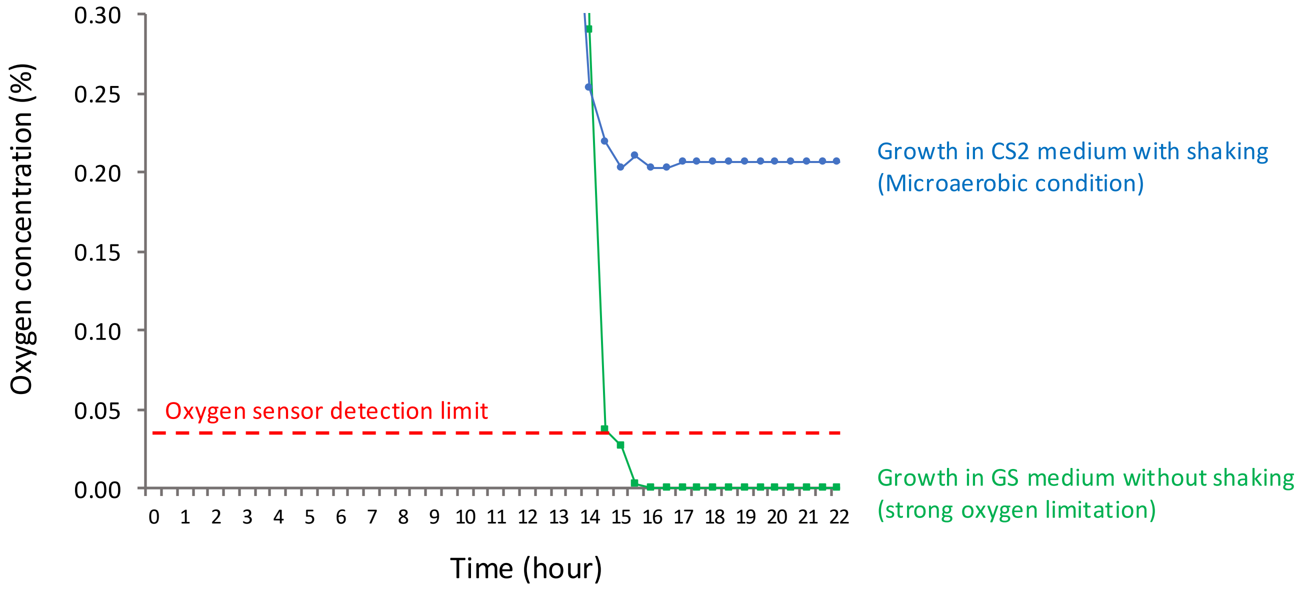

Supplementary Fig. 1. Comparison of oxygen concentration in CS2 and GS media

Evolution of oxygen concentration under microaerobic (blue curve) and strong oxygen limitation (green curve) are shown. The horizontal red dotted line shows our oxygen sensor detection limit (around 0.03 %) which limits our ability to precisely measure how low the oxygen concentration decreases in GS medium.

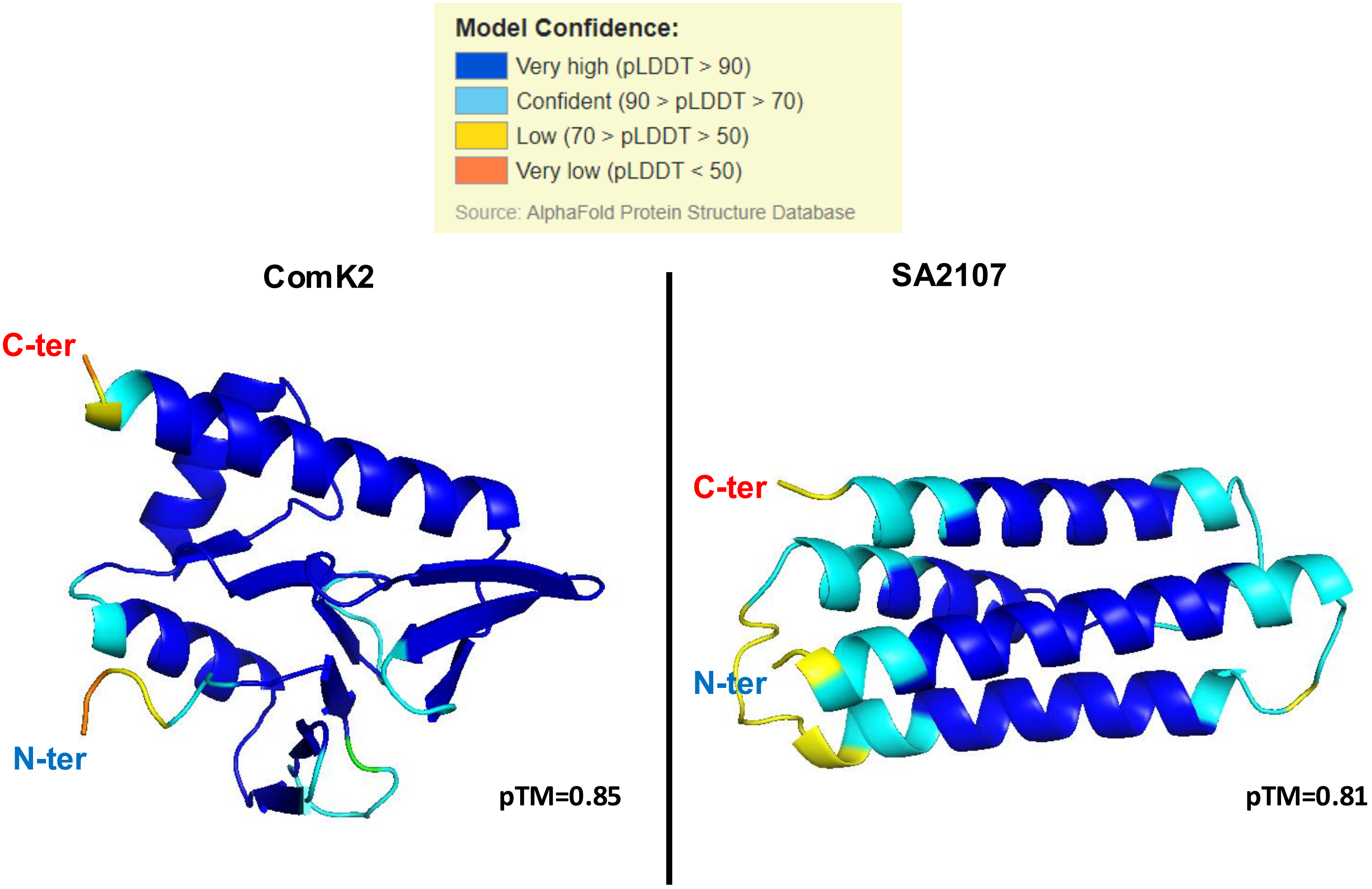

Supplementary Fig. 2. Predicted 3D structure of ComK2 and SA2107 (AlphaFold2)

We used AlphaFold2 to predict the three-dimensional structure of our proteins of interest (ComK2, SA_RS08990, left and SA2107, SA_RS12110, right). The models proposed here are visualized on the PyMol software, displaying the proteins in “cartoon” format (α-helices and β-sheets visible), and coloring them according to the plDDT statistic calculated by the algorithm. The plDDT (predicted local distance difference test) corresponds to a measure of confidence in the interpretation of the structure, by amino acids. AlphaFold2 also calculates a pTM-score (predicted Template Modeling score), represented by a number between 0 and 1, and measuring the accuracy of the entire structure.

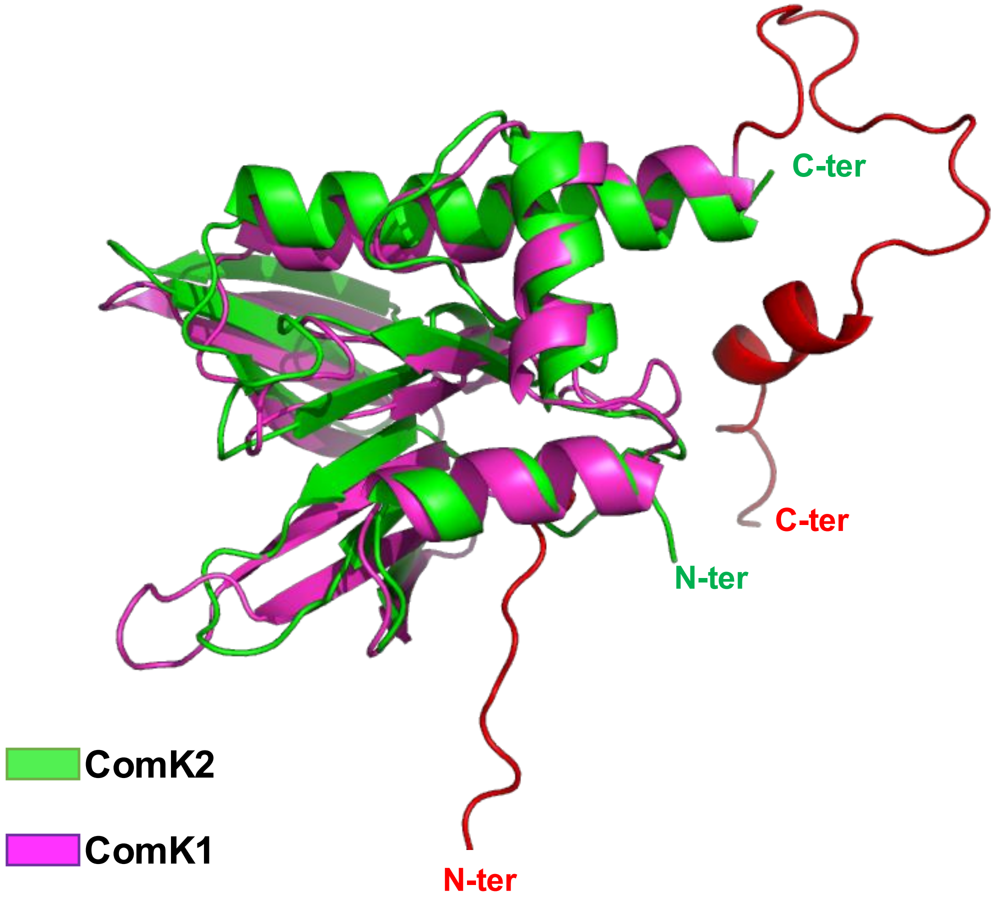

Supplementary Fig. 3. ComK1 and ComK2 predicted structures.

Superposition of ComK1 (in green) and ComK2 (in pink) predicted structures by AlphFold2. Extra amino acids present in N- and C-terminal of ComK1 are shown in red.

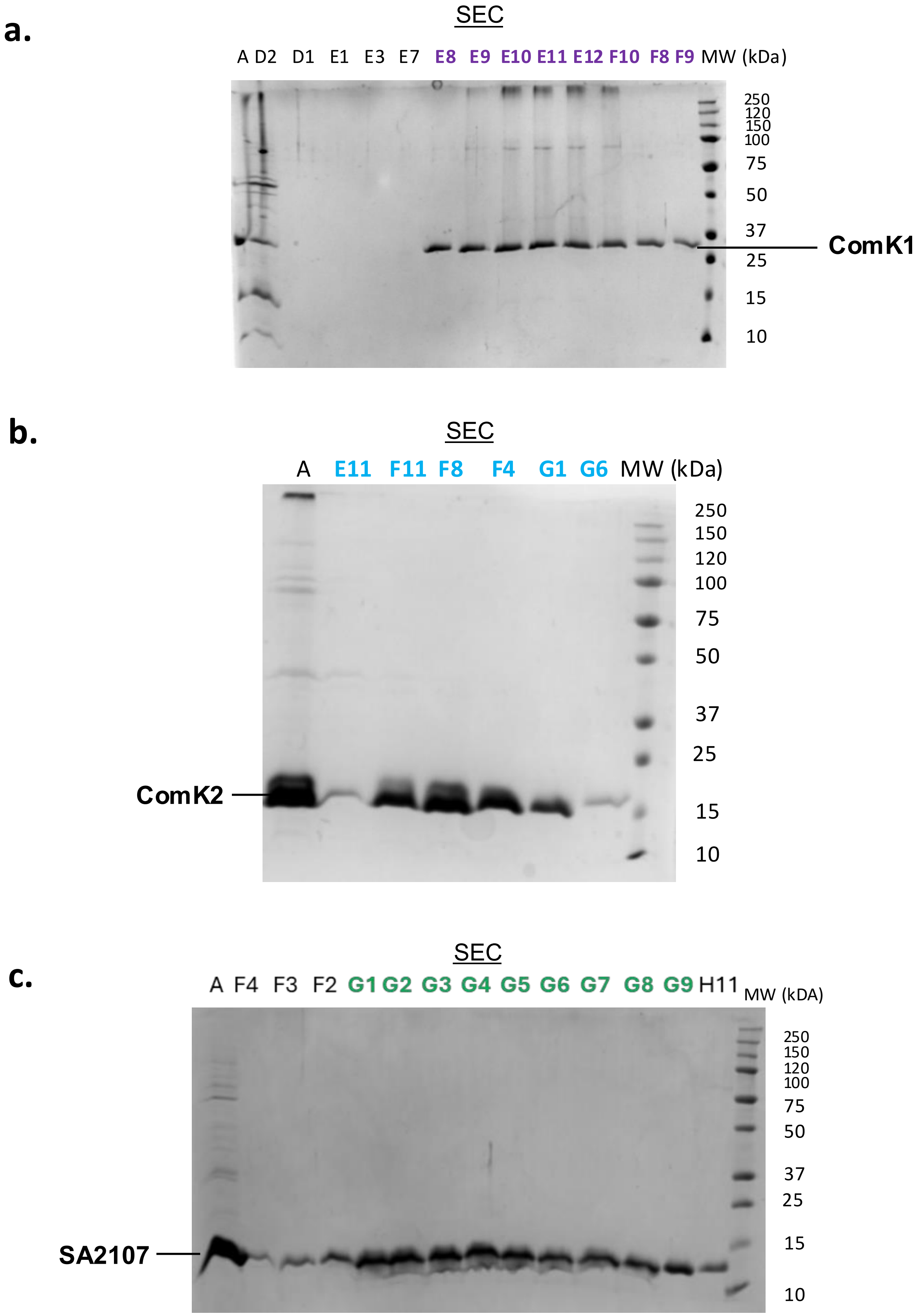

Supplementary Fig. 4. Purification of ComK1, ComK2 and SA2107

The different fractions obtained after purification by Size-Exclusion Chromatography (SEC) are shown in a SDS-PAGE gel. The "A" fraction correspond to the sample before injection in the apparatus. The other wells represents different fractions eluted in a 96-wells plate and containing the ComK1 (a), ComK2 (b), or SA2107 (c) proteins.

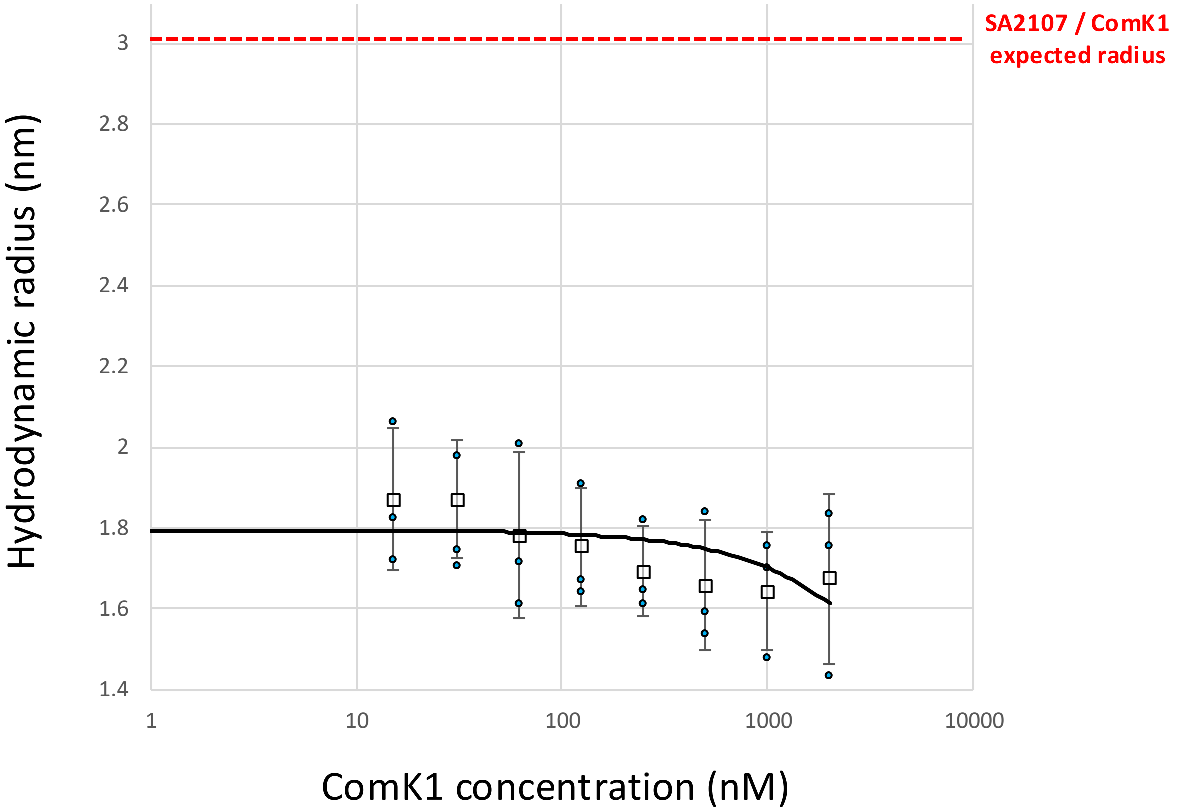

Supplementary Fig. 5. ComK1 does not interact with SA2107

Titration experiment of a fixed concentration of SA2107 (20 µM) mixed with ComK1 ranging from 15 nM to 2 µM. The expected radius of the potential SA2107/ComK1 complex is shown (red dotted line). SA2107 hydrodynamic radius is calculated based on three independent experiments (mean and SD). For each data point, individual hydrodynamic radius values are presented as blue circles.

The calculated hydrodynamic radius of SA2107 does not change, even at high ComK1 concentrations demonstrating that ComK1 and SA2107 do not interact.

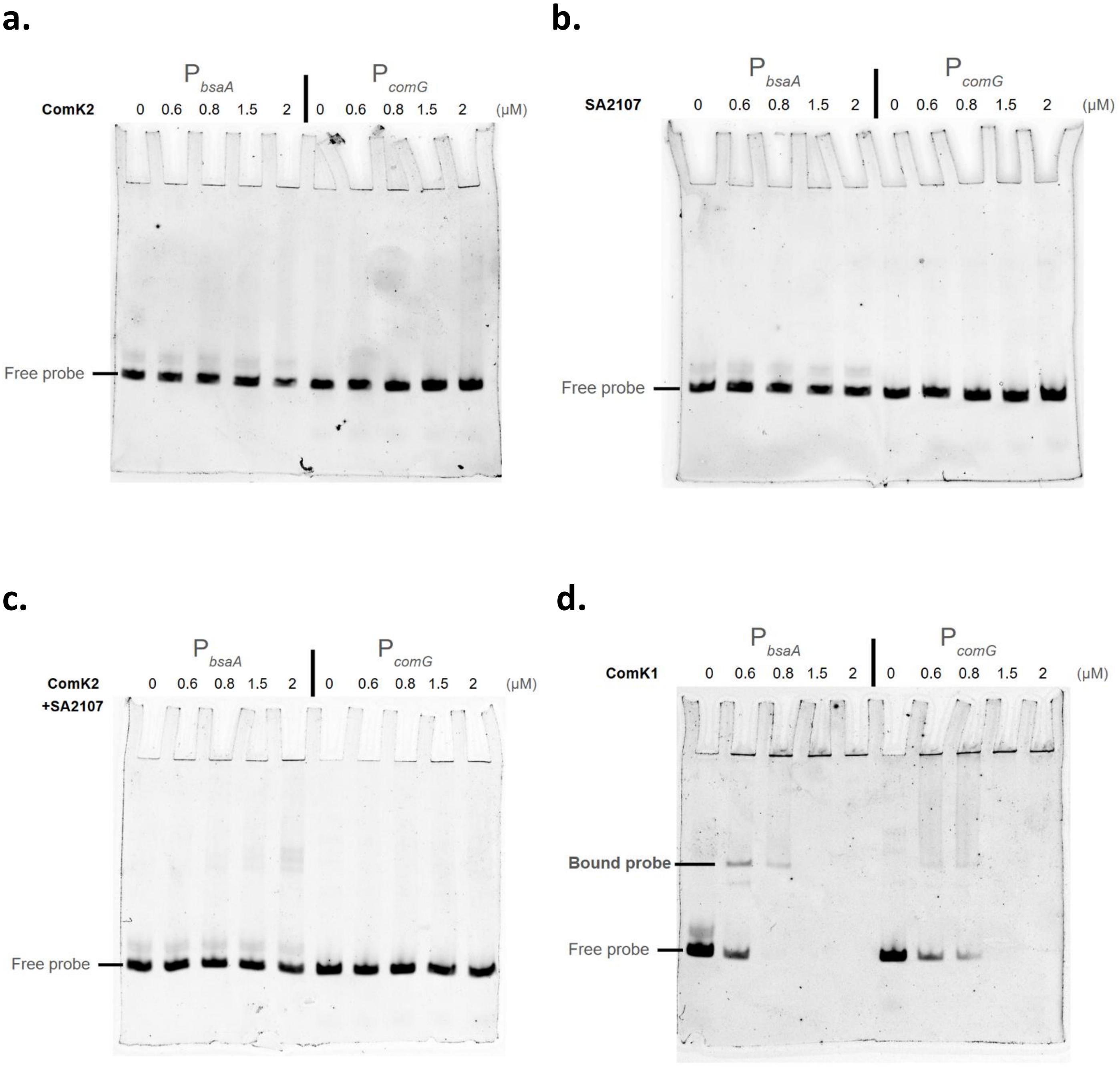

Supplementary Fig. 6. Electromobility Shift Assays to test the ability of ComK1, ComK2, SA2107 as well as the ComK2/SA2107 complex to bind the *comG* operon promoter.

Probes corresponding to the two promoters (P_{comG} and P_{bsaA} used here as a negative control) were tested. A range of proteins (ComK2, a; SA2107, b; ComK2 + SA2107, c; ComK1, d) ranging from 0 to 2 μM were tested on each probe. Free DNA and shifted bands were arrow-indicated.

Supplementary Tables

Model number	Random seed	average pLDDT	pTM-score	ipTM-score	Combined pTM-score
1	25271	90.64	0.89	0.88	0.882
4	25271	91.44	0.89	0.88	0.882
5	25271	90.03	0.89	0.86	0.866
3	25271	90.91	0.87	0.83	0.838
2	25271	90.54	0.86	0.79	0.804

Supplementary Table 1. Predicted 3D structure of ComK2 and SA2107 (AlphaFold2)

Statistical scores (see material and methods for details) of the best 5 predicted models of the ComK2-SA2107 complex by AlphaFold2.

Type of interaction	ComK2	SA2107
Hydrogen bonds	Ser 5	Asp 20
	Tyr 31	Asp 20
	Asn 4	Asn 92
	Asn 4	Thr 96
	Asp 3	Lys 23
	Ser 5	Lys 23
	His 28	Arg 9
	Ile 32	Gln 17
Salt bridge	Asp 3	Lys 23

Supplementary Table 2. Amino acids and types of interactions involved in the ComK2-SA2107 complex.

Different types of interactions highlighted between ComK2 and SA2107, and associated residues. Amino acids involved in hydrogen bonds or salt bridges were identified manually on PyMol (displaying amino acids less than 4 Å away from each other, that could form polar contacts at the interface between ComK2 and SA2107), then confirmed using the PISA tool (Proteins, interfaces, Structures and Assemblies, on the European Protein Data Bank).

Model number	Random seed	average pLDDT	pTM-score	ipTM-score	Combined pTM-score
3	83543	74.40	0.57	0.20	0.274
1	83543	73.54	0.56	0.14	0.224
2	83543	77.15	0.57	0.12	0.210
5	83543	73.12	0.56	0.12	0.208
4	83543	72.15	0.55	0.11	0.198

Supplementary Table 3. ComK1 does not seem to interact with SA2107.

Statistical scores (see material and methods for details) of the best 5 predicted models of the ComK1-SA2107 complex by AlphaFold2.

Strains	Genotype/Construction	Source
S. aureus		
**St012**	N315ex woφ	8
**St029**	N315ex woφ / pRIT-P_{comG}-*gfp*	8
**St050**	N315ex woφ / pRIT-P_{ssb}-*gfp*	13
**St037**	N315ex woφ Δ*comK1*	13
**St040**	Δ*comK1* / pRIT-P_{comG}-gfp	13
**St064**	Δ*comK1* / pRIT-P_{ssb}-*gfp*	13
**St038**	N315ex woφ Δ*comK2*	13
**St041**	Δ*comK2* / pRIT-P_{comG}-*gfp*	13
**St067**	Δ*comK2* / pRIT-P_{ssb}-*gfp*	13
**St045**	N315ex woφ Δ*sigH*	13
**St051**	Δ*sigH* / pRIT-P_{comG}-*gfp*	13
**St061**	Δ*sigH* / pRIT-P_{ssb}-*gfp*	13
**St039**	N315ex woφ Δ*sa2107*	This study
**St042**	Δ*sa2107* / pRIT-P_{comG}-*gfp*	This study
**St161**	Δ*comK2* - Δ*sa2107* / pRIT-P_{comG}-*gfp*	This study
**St145**	Δ*srrA* / pRIT-P_{comG}-*gfp*	13
**St147**	Δ*srrA* / pRIT-P_{ssb}-*gfp*	13
**St118**	N315ex woφ Δ*nreC*	13
**St158**	Δ*nreC* / pRIT-P_{comG}-*gfp*	13
**St177**	Δ*airR* / pRIT-P_{comG}-*gfp*	13
**St273**	Δ*nreC* – Δ*comK2* / pRIT-P_{com}G-*gfp*	13
**St197**	N315ex woφ pCN34	13
E. coli		
**ECN145**	pET21-*comK2*	This study
**ECN146**	pET29-*sa2107*	This study
S. cerevisiae		
**121**	empty pGBDU	19
**131**	empty pGAD	19
**YE008**	pGBDU-*comK2*	This study

Supplementary Table 4. Strains used in this study

Name	Nucleotide sequence	Descriptions
IM151	TAC ATG TCA AGA ATA AAC TGC CAA AGC	Used for verifying and sequencing the allelic replacement pIMAY constructs
IM152	AAT ACC TGT GAC GGA AGA TCA CTT CG	
Kpn I-SA2017-up-F	GCA GGT ACC CAT ATG ACA CTC CCA ATG C	To amplify upstream of sa2107 used for the deletion of *sa2017*
Sal I-SA2017-up-R	GCA GTC GAC GTA GAA GTA CCT CCA AAA ATC AAT	
EcoRI-SA21017-down-F	GCA GAA TTC GCC CAT TAA CCT ATT TTT CAT A	To amplify downstream of sa2107 used for the deletion of *sa2017*
Sac I-SA2107-down-R	GCA GAG CTC ACA ATA TTT GAT GCC TGT GCT A	
check-mutant-SA2107-F	TGA AAG TCA GTC GTA CTC GAC AT	To verify the *sa2107* deletion mutant
check-mutant-SA2107-R	GTT GCT CCC ATA TGC ATC TCA	
Sal I-comK2-F	GCA GCA GTC GAC TTG CAA GAC AAT TCT ACT AAA TAT CTA C	To amplify the *comK2* gene and clone it in the pGBDU
Bgl II-comK2-R	GCA GCA AGA TCT TTA ATT CGA AAT AGT ATT TTC AAT GAA GTG C	
NdeI-comK2-F	TTTTTCATATGCAAGACAATTCTAC	To amplify the *comK2* gene, tagged with 6 His and clone it in the pET21
Xho-comK2-His-R	TTTTTCTCGAGTCAGTGATGGTGATGGTGATGATTCGAAATAGTA	
NdeI-His-SA2107-F	TTTTTCATATGCATCACCATCACCATCACATAAACGAAACTAAG	To amplify the *sa2107* gene, tagged with 6 His and clone it in the pET29
Xho-His-SA2107-R	TTTTTCTCGAGTCAATGGGCAAATTTAATTG	

Supplementary Table 5. Primers used in this study
